# Two Toxins, One Disorder: Neurophysiological and Clinical Effects in Caput-Type of Cervical Dystonia at Waning and Peak Response Phases of BoNT-A Therapy

**DOI:** 10.3390/toxins18070277

**Published:** 2026-06-24

**Authors:** Artur Drużdż, Małgorzata Dudzic, Igor Bednarski, Anna Sowińska, Natalie Górna, Agnieszka Przystańska

**Affiliations:** 1 Faculty of Medicine, Prince Mieszko I Poznan Medical University of Applied Sciences, 60-320 Poznań, Poland; 2 Department of Neurology, Municipal Hospital, 61-285 Poznań, Poland; mdudzic@szpital.strusia.poznan.pl; 3 Department of Neurology, Central University Hospital, 92-213 Łódź, Poland; igorbednarski@gmail.com; 4 Department of Computer Science and Statistics, Poznań University of Medical Sciences, 61-701 Poznań, Poland; ania@ump.edu.pl; 5 Department of Orthodontics and Temporomandibular Disorders, Poznań University of Medical Sciences, 60-812 Poznań, Poland; natalie.nejsi@gmail.com; 6 Department of Anatomy, Poznań University of Medical Sciences, 60-781 Poznań, Poland; aprzyst@ump.edu.pl

**Keywords:** botulinum toxin (BoNT-A), cervical dystonia, Col-Cap concept, F-wave, cutaneous silent period (CSP), onabotulinumtoxinA (ONA), abobotulinumtoxinA (ABO)

## Abstract

Botulinum toxin type A (BoNT-A) is an established treatment for cervical dystonia (CD), but objective neurophysiological markers across the injection cycle and between preparations are not well defined. We assessed afferent conduction (captured by CSP parameters), efferent conduction (captured by F-wave parameters) and clinical severity (TWSTRS) in 28 patients with caput-type cervical dystonia during waning (>14 weeks post-injection) and peak (4–6 weeks) phases, comparing onabotulinumtoxinA (ONA) and abobotulinumtoxinA (ABO). F-wave measures changed only modestly: F–M latency increased with ONA, while F-wave persistence decreased with ABO. In contrast, CSP measures consistently increased at peak in both groups (CSP end latency and CSP duration; both *p* ≤ 0.001). Overall, BoNT-A treatment phase is better reflected by CSP-derived inhibitory measures than by F-wave indices. TWSTRS improved at peak for both toxins, with no difference between ONA and ABO in clinical change (ΔTWSTRS *p* = 0.5514).

## 1. Introduction

Cervical dystonia (CD) manifests as involuntary, persistent, or intermittent contractions of cervical muscles, which lead to atypical head and neck postures or movements [1]. The Col-Cap concept—an anatomical and functional framework used for classifying dystonic patterns and guiding treatment targeting—distinguishes between the muscles located above the C3 vertebra that affect the postures of the head (termed caput) and those below the C2 vertebra that affect the postures of the neck (termed collis) [2].

In Poland, the therapeutic landscape for CD is dominated by two botulinum neurotoxin type A (BoNT-A) formulations: onabotulinumtoxinA (ONA; Botox^®^) and abobotulinumtoxinA (ABO; Dysport^®^), both of which exert their clinical effects via cleavage of SNAP-25, thereby inhibiting presynaptic acetylcholine release at the neuromuscular junction and producing reversible chemodenervation with subsequent muscle relaxation [3]. Therefore, on mechanistic grounds alone, these formulations might be expected to show broadly similar clinical effects.

However, ONA and ABO are not identical products. Both are formulated as high-molecular-weight complexes in which the 150 kDa neurotoxin is non-covalently bound to neurotoxin-associated proteins (NAPs), but their compositions differ: ONA forms a homogeneous 900 kDa complex, whereas ABO consists of a heterogeneous mixture predominantly under 500 kDa [3]. These formulation-level differences could plausibly translate into meaningful biological effects, potentially influencing efficacy and the duration of action in CD.

Although ONA and ABO have been compared extensively, most clinical comparisons relied on subjective outcome measures, leaving uncertainty about how molecular and formulation differences map onto objective, in vivo neurophysiological effects. Emerging evidence suggests that outcomes such as duration of effect may be toxin-dependent, supporting the need for mechanistic studies to optimise therapeutic strategies and refine evidence-based practice [4].

The F-wave and the cutaneous silent period (CSP) are two extensively utilised, non-invasive neurophysiological measures that provide insights into distinct, yet complementary, dimensions of nervous system excitability and inhibitory function. The F-wave itself constitutes a late response elicited by applying supramaximal electrical stimulation to a motor nerve. This particular neurophysiological signal serves as an indicator of both anterior horn cell excitability and proximal motor conduction [5,6].

Abnormalities of F-wave parameters have been reported across a range of neurological disorders, including dystonia and conditions affecting both central and peripheral motor pathways. In primary torsion dystonia, the frequency of occurrence and mean amplitudes of the F-wave recorded from both the median and ulnar nerves were higher than normative values [7]. In amyotrophic lateral sclerosis (ALS), increased mean, minimal, and maximal F-wave latencies, as well as increased chronodispersion, were reported [8,9,10]. Taken together, these observations support the sensitivity of F-wave metrics to clinically relevant changes in efferent motor neuron function. In this study, F-wave analysis was used as an index in the assessment of efferent pathway modulation during BoNT-A treatment.

Conversely, the CSP represents a temporary cessation of voluntary muscle contraction that occurs subsequent to a painful skin stimulus. This response is understood to be an inhibitory phenomenon, originating from both spinal and supraspinal levels, with partial mediation by A-delta fibres [11]. Prolonged CSP durations have been reported in other forms of focal dystonia, for example, in studies conducted by Pullman et al. [12]. CSP measures were included in this study to provide insight into afferent and central pathway modulation in BoNT-A injection cycle.

Together, F-wave and CSP measures provide complementary indices of efferent motor excitability and afferent-driven inhibitory processing, respectively, and can therefore be used to characterise neurophysiological dynamics at maximum and minimum effect of BoNT-A. However, despite extensive clinical comparisons between ONA and ABO, few studies have directly examined whether these formulations differ in their effects on such neurophysiological markers across the injection cycle. In our previous work [13], we have observed phase-dependent changes in F-wave and CSP parameters, however the sample size was smaller and toxin preparation was not controlled, warranting a formulation-specific comparison.

Accordingly, this study compares objective neurophysiological markers (F-wave and CSP parameters) and clinical severity (the Toronto Western Spasmodic Torticollis Rating Scale, TWSTRS) in patients with caput-type CD treated with ONA versus ABO, assessed at both the waning and peak phase of the therapeutic response. It has been hypothesised that, despite a shared SNAP-25–mediated mechanism, ONA and ABO would show measurably different neurophysiological and clinical profiles across the treatment cycle, with differences most apparent when contrasting peak and waning phases, consistent with differential modulation of excitatory/inhibitory circuitry and/or effect duration. An additional objective was to explore novel neurophysiological parameters that may be useful for assessing afferent (sensory) pathways and conduction within structures of the central nervous system.

## 2. Results

As shown in Table 1, neurophysiological outcomes at the peak response phase differed by toxin preparation. F–Mlat measures changed in the ONA group, from 20.0 ± 2.1 ms to 20.6 ± 2.1 ms (*p* = 0.049). No statistically significant differences were observed in the remaining measured or calculated F-wave and M-wave parameters. In the ABO group (Table 1), we also observed only one change in the F-wave parameters, with F-wave persistence decreasing from 16.3 ± 2.4 responses to 15.1 ± 2.0 responses (*p* = 0.002).

CSP measures increased after both preparations. The administration of ONA was associated with highly significant increases in CSP end latency (CSPe), from 118.5 ± 9.1 ms to 130.7 ± 6.2 ms (*p* < 0.001), CSP duration (CSPd), from 39.6 ± 5.9 ms to 50.9 ± 7.3 ms (*p* < 0.001).

Similarly, the administration of ABO resulted in significant increases in CSP end latency (CSPe), from 116.7 ± 11.1 ms to 124.2 ± 8.0 ms (*p* = 0.002) and CSP duration (CSPd), from 39.5 ± 5.1 ms to 47.0 ± 6.4 ms (*p* = 0.001).

**Figure 1:**
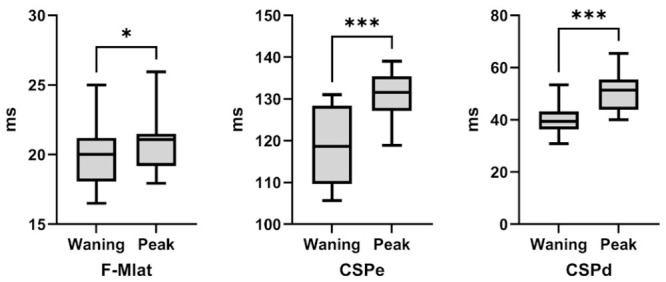
Significant neurophysiological effects of onabotulinumtoxinA (ONA). Data presented as box-whisker plots with medians (horizontal line within box), interquartile range (box) and min–max values (whiskers). Asterisks above plots indicate the level of statistical significance: * *p* < 0.05, *** *p* < 0.001.

**Figure 2:**
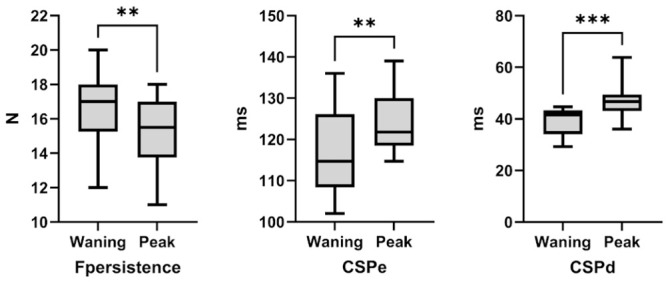
Significant neurophysiological effects of abobotulinumtoxinA (ABO). Data presented as box-whisker plots with medians (horizontal line within box), interquartile range (box) and min–max values (whiskers). Asterisks above plots indicate the level of statistical significance: ** *p* < 0.01, *** *p* < 0.001.

We did not find any significant changes in CSP onset latency (CSPo). Finally, clinical outcomes improved substantially in both groups, with total TWSTRS decreasing after ONA (33.2 ± 9.8 → 15.9 ± 5.3, *p* < 0.001) and after ABO (40.7 ± 6.4 → 25.3 ± 6.4, *p* < 0.001). Significant neurophysiological effects of both toxins are illustrated in Figure 1 and Figure 2.

In between-group comparisons of absolute TWSTRS scores (Table 1), statistically significant differences were found in the waning phase (*p* = 0.025), indicating that baseline clinical severity was not fully homogeneous. In the peak phase, total TWSTRS scores also differed between groups (*p* < 0.001), However, this difference reflects group-level differences in absolute scores at that time point rather than a statistically significant difference in treatment-related change, which is supported by the analysis of change between time points (Δ), as shown in Table 2.

**Figure 3:**
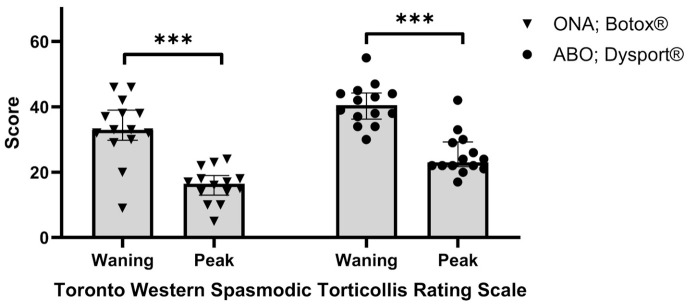
Changes in Toronto Western Spasmodic Torticollis Rating Scale (TWSTRS) in both ONA (downward triangles) and ANA (points) groups. Data presented as columns with medians (columns), interquartile range (whiskers). Asterisks above plots indicate the level of statistical significance: *** *p* < 0.001.

The mean reduction in total TWSTRS score was −17.3 ± 9.6 points in the ONA group and −15.4 ± 6.3 points in the ANA group, indicating a substantial clinical benefit in both cohorts, however the magnitude of improvement did not differ significantly between groups (*p* = 0.55), suggesting comparable clinical response when changes in TWSTRS scores were considered. Changes in TWSTRS are shown in Figure 3.

## 3. Discussion

This study evaluated whether two preparations of BoNT-A (ONA and ABO) differ in their neurophysiological and clinical effects at the waning and peak phases of treatment in patients with caput-type CD. Overall, F-wave indices showed limited modulations, whereas CSP-related measures were more robustly altered by both preparations, particularly at peak response. TWSTRS indicated clinical improvement in the peak response phase for both toxins. Together, these phase-dependent findings suggest that BoNT-A treatment is accompanied by measurable changes in neurophysiological markers, with the clearest effects captured by inhibitory measures.

With respect to F-wave parameters, administration of onabotulinumtoxinA (ONA) was associated with a prolongation of F–M interlatency, whereas abobotulinumtoxinA (ABO) treatment was associated with a substantial decrease in F-wave persistence. The toxins affected individual and disparate F-wave parameters, which does not constitute a comprehensive effect on F-wave parameters or on the peripheral motor neuron. Since F-wave parameters are used for assessing motor conduction and spinal motoneuronal excitability, the modest and selective nature of our findings in the present study suggests weak influence of BoNT-A on the efferent conduction in the peripheral motor neuron across the injection cycle, at least as captured by our protocol.

The existing literature does not provide direct comparisons of BoNT-A preparations’ impact on F-wave measures. One study by Wohlfarth et al. [14] concluded that ABO was the causative agent in prolonging F-wave latency by 1–3 ms and decreasing F-wave persistence decreased by about 20% in focal dystonia (cervical dystonia and writer’s cramp). This is partially consistent with our results (showing decreased F-wave persistence in the ONA group) and the results of our previous study [13], which found F–M interlatency prolongation and F-wave persistence decrease following a treatment with BoNT-A (toxin formulation was not a controlled variable in that study).

This contrasts with the more pronounced phase-dependent effects observed in CSP measures. After administration of ONA and ABO, substantial prolongation of CSP end latency (CSPe) and CSP duration (CSPd), most evident during the peak response phase, was observed. The fact that these CSP parameters were significantly shorter in the waning phase may corroborate previous reports indicating loss of inhibition (considered a direct contributor to excessive muscle activity) and sensorimotor disturbances consistent with the current conceptualisation of dystonia as a network disorder.

Kofler et al. [15] found CSP to be highly sensitive to intramedullary spinal cord pathology, especially in cases involving the spinothalamic pathways. These findings may lend support to the notion that the abnormalities identified in our cohort likewise reflect a central component, although one likely attributable to functional rather than structural mechanisms.

Clinically, both preparations were associated with a clear improvement in dystonia severity, as reflected by lower total TWSTRS scores during the peak phase compared with the waning phase. Although differences in absolute TWSTRS scores were observed between groups at both time points, these likely reflect baseline heterogeneity rather than a true difference in therapeutic response. Importantly, the magnitude of clinical improvement, as reflected by the difference between time points, was comparable between groups, supporting the interpretation that both preparations produced a similar clinical benefit. While peak-phase improvement is consistent with previous reports demonstrating beneficial effects of BoNT-A on TWSTRS outcomes in CD [16,17], the differences between the two toxins, as observed in absolute TWSTRS scores, should be interpreted with caution, as they may reflect baseline heterogeneity, dose-conversion differences, and inter-individual variability in treatment response rather than true differences in efficacy. Accordingly, replication in larger and methodologically standardised studies is needed.

Taken together, our findings suggest a convergence between neurophysiological and clinical effects across treatment phases. The peak phase was characterised by prolongation of CSP-derived measures (including indices interpreted as reflecting central inhibitory processing) and improvement in TWSTRS. In contrast, during the waning phase, CSP measures were shorter and clinical benefit was reduced, consistent with the expected time course of BoNT-A efficacy. This phase-dependent coupling supports the utility of CSP-related measures as potential objective markers of treatment state in caput-type CD.

We acknowledge several limitations within this study. For example, the number of participants was modest, which limits precision, and the study was conducted at a single centre, affecting generalisability. It is possible that studies with larger sample sizes would provide sufficient statistical power to detect differences between BoNT-A formulations that were not evident in the present analysis. Also, neurophysiological testing was confined to a single nerve, therefore, the extent to which the observed F-wave and CSP changes reflect broader sensorimotor network physiology in CD remains uncertain. In addition, participants had prior BoNT-A exposure, which may influence baseline neurophysiological measures through long-term peripheral and central adaptations. Consequently, the findings may not generalise to treatment-naïve patients and complicate interpretation of absolute values as disease markers. Future studies should use standardised injection protocols, ideally in randomised designs, with longitudinal follow-ups across multiple injection cycles.

## 4. Materials and Methods

The Neurology Department of the Municipal Hospital in Poznań was the site for this prospective cohort study, which took place from 17 July 2023 to 20 December 2024. Prior to inclusion, written informed consent was secured from every participant. The study adhered to the principles set forth in the Declaration of Helsinki. Ethical clearance was provided by the Bioethics Committee of the Poznan University of Medical Sciences on 29 March 2023 (approval no. 525/2023). It was subsequently listed in the UK Clinical Study Registry (ISRCTN11389213) on 22 October 2025.

### 4.1. Participants

Twenty-eight individuals diagnosed with cervical dystonia (CD), based on the 2023 criteria set out by Albanese et al. [18], were enrolled in this study. Recruitment occurred via consecutive sampling from an existing patient cohort. Eligibility required patients to exclusively exhibit caput patterns, according to the Col–Cap concept [1], and to have received treatment at the facility through the National Health Fund (NFZ) therapeutic programme for at least 1.5 years before inclusion. Ten patients who participated in our previous study [13] provided consent for continued participation and were therefore included in the new cohort.

Participants were excluded if they had peripheral nervous system damage secondary to metabolic disorders, toxic neuropathies, rheumatologic diseases, inflammatory neuropathies, or compressive (entrapment) neuropathies. Additional disqualifying factors included administration of any pharmaceutical agents recognised for their impact on nerve conduction.

Prior to inclusion, all participants underwent a comprehensive screening protocol. This involved a thorough neurological assessment (encompassing evaluation of pain, light touch, vibration sense, and tendon reflexes), brain and cervical spine imaging tests, and laboratory tests. These initial tests collectively demonstrated the absence of any pathology. Furthermore, at the point of enrolment, three specialised clinicians independently verified the presence of stable caput patterns and ensured the complete absence of tremulous, non-rhythmic, phasic, or myoclonic movements. An overview of the identified caput pattern distribution is presented below:

- TCap/LCap: 6 ONA, 6 ABO
- TCap/LCap/ACap: 3 ONA, 2 ABO
- TCap/LCap/RCap: 2 ONA, 3 ABO
- TCap/ACap: 2 ONA, 2 ABO
- TCap/RCap: 1 ONA, 1 ABO

Participants were allocated to two groups according to the BoNT-A formulation used in their treatment. The onabotulinumtoxinA group (ONA; Botox^®^) included 11 females and 3 males (mean age 53.8 ± 8.2 years) and received a mean dose of 224.9 ± 34.19 units. The mean duration of BoNT-A treatment in this group was 7.3 ± 3.1 years (26.2 ± 10.2 treatment cycles). The abobotulinumtoxinA group (ABO; Dysport^®^) included 11 females and 3 males (mean age 53.3 ± 7.6 years) and received a mean dose of 696.36 ± 170.42 units. The mean treatment duration in this group was 6.9 ± 2.9 years (25.7 ± 10.5 treatment cycles). We used ultrasound to inject BoNT-A into muscle selected according to the Col–Cap concept [1,2].

Assessments were performed at two timepoints. The first, at >14 weeks after BoNT-A administration, represents the waning phase, defined as diminished therapeutic benefit with participants reporting an approximate 50% worsening of symptoms. The second assessment was conducted 4–6 weeks after injection, reflecting the peak treatment effect. All 28 participants completed both rounds, with no dropouts. No adverse events were observed.

### 4.2. Neurophysiological Assessments

All neurophysiological testing was performed at a single centre (Neurology Department, Municipal Hospital in Poznań, Poland) by trained staff, under standardised environmental conditions.

#### 4.2.1. General Testing Conditions and Equipment

The testing sessions were managed by a technician with a decade of experience. Oversight and subsequent interpretation of all results were provided by a neurophysiologist (MD, PhD) with expertise in the excess of 25 years. To mitigate potential bias, investigators involved in either the testing procedures or data interpretation had no affiliation with the National Health Fund (NFZ) therapeutic programme from which the participants were enrolled.

All tests were conducted in a quiet room, where the temperature was consistently held between 22 and 24 °C, and participants’ skin temperature was kept at or above 32 °C. For examination, individuals were positioned supine on a medical couch, ensuring their upper limbs were relaxed and properly supported. Each testing session spanned approximately 30 min, totalling 60 min per participant across the two timepoints.

A 3-channel electromyography (EMG) system was used to record signals, specifically the Dantec Keypoint G4 (type 9031A070101) from Natus Manufacturing Ltd., Galway, Ireland. This system incorporated the manufacturer’s F-wave and Motor Nerve Conduction software applications. The electrode configuration comprised a stimulating electrode (reference no. 9013L0362) from Natus Manufacturing Ltd., Galway, Ireland, a recording electrode (reference no. 9013S0242) from Ambu A/S, Ballerup, Denmark and a ground electrode (reference no. GDRGP0450326) from Spes Medica S.r.l., Genova, Italy.

#### 4.2.2. M-Wave and F-Wave Protocol

The M-wave and F-wave protocol was adapted from Fisher et al. [6] and Puksa et al. [19]. Motor nerve conduction and F-wave recordings were performed on the right upper limb (all participants were right-handed), with responses recorded from the abductor pollicis brevis (APB) muscle.

Surface electrodes were set in a belly–tendon configuration, i.e., the active electrode was positioned over the abductor pollicis brevis (APB) muscle belly, and the reference electrode was placed on its distal tendon. The ground electrode was placed on the dorsum of the hand. Stimulation of the median nerve occurred at the wrist, utilising a superficial bipolar electrode. This stimulation employed a supramaximal intensity, typically set at 20–30% beyond the threshold required to evoke a maximal compound muscle action potential (CMAP), with a pulse duration of 0.2 ms. Twenty individual stimuli were delivered at a frequency of 0.5 Hz.

Regarding the F-wave, the band-pass filtering was configured at 20 Hz to 10 kHz, the amplifier gain at 0.5 mV/division, and the sweep speed at 5 ms/division. For M-wave acquisition, identical band-pass filtering (20 Hz–10 kHz) was applied, the amplifier gain was set to 5 mV/division, and the sweep speed at 5 ms/division.

The following electrophysiological parameters were measured:

- Fmin (ms)—minimal latency of all F-wave responses received
- Fmax (ms)—maximal latency of all F-wave responses received
- Fmean (ms)—calculated as Fmin + (Fmax − Fmin)/2
- Fchronodisp (ms)—chronodispersion of F-waves; interval between Fmin and Fmax
- Fduration (ms)—mean duration of each F-wave response
- Fpersistence (*n*)—number of F-wave responses observed in 20 stimulations
- Fampl (mV)—mean amplitude of all F-wave responses
- Mlat (ms)—latency of M-wave
- Mampl (mV)—maximal amplitude of M-wave

The following ratios were analysed:

- F/Mampl—F/M amplitude ratio, calculated as mean F-wave amplitude ÷ maximal M-wave amplitude
- F–Mlat (ms)—F–M latency difference, calculated as Fmin − Mlat

#### 4.2.3. CSP Protocol

The CSP protocol was derived from the ones previously published by Kofler et al. [20], Tiric-Campara et al. [21], Bölük et al. [22] and Neves et al. [23].

CSP recordings were captured from the abductor pollicis brevis (APB) of the right (dominant) hand. Surface electrodes were applied in a belly–tendon configuration: the active electrode rested on the muscle belly, the reference electrode on the distal tendon, and the ground electrode was positioned on the dorsum of the hand. During this process, skin impedance was consistently maintained below 5 kΩ.

The digital nerve of the right index finger was stimulated using a bipolar surface stimulating electrode. Throughout each recording session, participants were instructed to sustain a steady isometric contraction of the APB, aiming for about 40–50% of their maximal effort.

Stimulus intensity was set at 20× the sensory threshold (ST). Mean ST was 1.80 ± 0.54 mA (mean ± SD), with pulse duration of 0.2 ms. EMG signals were recorded with band-pass filters set at 20 Hz–5 kHz, sweep speed at 20–50 ms/division, and sensitivity at 0.5–1 mV/division. Each participant received 10 stimuli at irregular, randomised intervals to minimise neural adaptation and habituation to repeated stimulation.

The CSP was visually identified as a suppression of ongoing voluntary EMG activity following the afferent stimulus. The precise temporal boundaries of CSP were determined by monitoring EMG amplitude: the CSP onset when the EMG signal dropped below 80% of its pre-stimulus baseline and the offset when it subsequently recovered to exceed that 80% threshold. A minimum of five recordings per participant were selected for analysis. The following CSP parameters were recorded:

- CSPo (ms)—latency at the beginning of muscle activity suppression
- CSPe (ms)—latency at the onset of renewed muscle activity
- CSPd (ms)—duration of the silent period, calculated as CSPe − CSPo.

### 4.3. Clinical Assessment

Clinical symptoms of cervical dystonia were assessed using the Toronto Western Spasmodic Torticollis Rating Scale (TWSTRS). This instrument is widely used and has been validated for the clinical assessment of cervical dystonia severity. It consists of three subscales: motor symptom severity (including the range and duration of head and neck movements, effectiveness of sensory tricks, and shoulder elevation), disability in activities of daily living, and pain assessment [24,25]. For statistical analyses, the total TWSTRS score was used. TWSTRS was administered at both study timepoints (waning and peak), on the same day as the neurophysiological assessment before the F-/M-wave and CSP recording. All TWSTRS assessments were conducted by an experienced neurologist from another centre.

### 4.4. Data Handling

Data were collected in accordance with ethical standards and Good Clinical Practice (GCP). Neurophysiological recordings were obtained by a qualified technician under the supervision of a neurophysiologist (licence no. EMG-41) who verified recording settings. Data were entered into standardised forms and stored in a restricted institutional database. Prior to analysis, datasets were anonymised and independently reviewed by a second neurophysiologist, who cross-checked them against source EMG data as needed. This reviewer had no contact with study participants. Data handling complied with institutional policy and GDPR requirements and was subject to regular audits (most recent audit: 14–18 July 2025).

### 4.5. Statistical Analysis

Statistical analysis was performed using the Statistica 13 software (StatSoft, Tulsa, OK, USA). Continuous variables were presented using mean and standard deviation (SD), as well as median with interquartile range (IQR). Distribution of the variables was assessed using the Shapiro–Wilk test. To compare differences between dependent groups, Student’s *t*-test or the Wilcoxon signed rank test were used, while in the case of independent groups, Student’s *t*-test with Welch’s correction or Mann–Whitney U test was employed. To assess the intervention effect, change scores (Δ = valueafter − valuebefore) were calculated for each participant and subsequently compared between groups, allowing direct evaluation of intervention-related changes and reducing the influence of baseline variability. Given that only two measurement time points were available, the analysis of change scores represents a valid alternative to repeated measures ANOVA providing information related to the assessment of the group × time interaction. A *p*-value below 0.05 for all comparisons was deemed significant.

## Figures and Tables

**Table 1:** F-wave, M-wave, CSP parameters and Toronto Score at the waning and the peak response phases of treatment with onabotulinumtoxinA (ONA) and abobotulinumtoxinA (ABO) and direct comparison of changes between groups. (a—*t*-test; b—Wilcoxon; c—Mann–Whitney).

	ONA (*n* = 14)			ABO (*n* = 14)			ONA vs. ABO	
Parameter (Unit)	Waning Mean ± SD Median (25–75 P)	Peak Mean ± SD Median (25–75 P)	*p*-Value	Waning Mean ± SD Median (25–75 P)	Peak Mean ± SD Median (25–75 P)	*p*-Value	Waning *p*-Value	Peak *p*-Value
Fmin (ms)	23.6 ± 2.0	24.2 ± 1.8	0.054 ^a^	23.6 ± 1.5	24.1 ± 1.6	0.1236 ^a^	0.946 ^a^	0.872 ^a^
	23.7 (21.9–24.6)	24.4 (22.7–25.0)		24.1 (22.1–25.1)	24.4 (23.4–25.1)			
Fmax (ms)	28.6 ± 2.1	29.5 ± 2.4	0.150 ^a^	29.8 ± 2.1	30.1 ± 2.1	0.448 ^a^	0.146 ^a^	0.474 ^a^
	28.8 (27.3–29.7)	29.5 (27.7–30.4)		29.8 (28.9–31.2)	30.0 (29.0–32.0)			
Fmean (ms)	26.1 ± 1.8	26.8 ± 1.8	0.059 ^a^	26.7 ± 1.4	27.1 ± 1.3	0.143 ^a^	0.347 ^a^	0.659 ^a^
	26.5 (24.5–27.7)	26.8 (25.2–27.6)		26.4 (25.6–28.2)	26.8 (26.2–28.4)			
Fchronodisp (ms)	5.0 ± 2.0	5.2 ± 2.5	0.647 ^a^	6.24 ± 2.44	6.0 ± 2.7	0.345 ^b^	0.153 ^a^	0.376 ^c^
	4.9 (4.1–5.7)	5.4 (4.7–5.8)		6.6 (4.7–7.6)	6.9 (4.8–7.8)			
Fduration (ms)	12.5 ± 1.2	13.4 ± 1.5	0.089 ^a^	12.3 ± 1.9	12.7 ± 2.3	0.580 ^a^	0.676 ^a^	0.390 ^a^
	12.6 (11.4–13.2)	13.6 (12.9–14.1)		12.1 (10.7–13.4)	12.9 (12.0–14.1)			
Fpersistence (*n*)	16.9 ± 3.0	16.3 ± 2.4	0.529 ^b^	16.3 ± 2.4	15.1 ± 2.0	0.002 ^a^	0.511 ^c^	0.186 ^a^
	17.0 (14.0–20.0)	17.0 (15.0–18.0)		17.00 (16.0–18.0)	15.5 (14.0–17.0)			
Fampl (µV)	0.3 ± 0.1	0.3 ± 0.1	0.152 ^b^	0.4 ± 0.1	0.3 ± 0.1	0.064 ^a^	0.227 ^c^	0.168 ^a^
	0.3 (0.3–0.4)	0.2 (0.2–0.3)		0.4 (0.3–0.4)	0.3 (0.3–0.4)			
F/Mampl	4.3 ± 1.4	3.7 ± 1.8	0.433 ^a^	5.3 ± 2.5	4.6 ± 2.0	0.056 ^b^	0.218 ^a^	0.246 ^a^
	4.5 (3.6–5.2)	3.7 (2.6–4.4)		4.9 (3.9–5.8)	4.4 (3.9–5.3)			
F–Mlat (ms)	20.00 ± 2.1	20.61 ± 2.1	0.049 ^a^	19.8 ± 1.7	20.3 ± 1.7	0.126 ^a^	0.770 ^a^	0.194 ^a^
	20.0 (18.2–21.2)	21.1 (19.2–21.4)		20.3 (18.2–20.9)	20.7 (19.3–21.2)			
CSPo (ms)	79.0 ± 10.7	79.8 ± 10.2	0.802 ^a^	77.2 ± 12.4	77.2 ± 11.3	0.997 ^a^	0.693 ^a^	0.535 ^a^
	79.7 (69.1–87.8)	80.90 (70.0–88.0)		73.8 (69.7–87.8)	73.8 (71.3–90.0)			
CSPe (ms)	118.5 ± 9.1	130.7 ± 6.2	<0.001 ^a^	116.7 ± 11.1	124.2 ± 8.0	0.002 ^a^	0.648 ^a^	0.023 ^a^
	118.7 (110.0–128.0)	131.6 (128.0–134.8)		114.7 (109.2–124.5)	121.8 (118.5–129.0)			
CSPd (ms)	39.6 ± 5.9	50.9 ± 7.3	<0.001 ^a^	39.5 ± 5.1	47.0 ± 6.4	<0.001 ^b^	0.571 ^a^	0.140 ^a^
	39.4 (37.6–43.2)	51.4 (44.0–55.4)		41.6 (34.0–43.2)	46.7 (43.0–49.3)			
Toronto score	33.2 ± 9.8	15.9 ± 5.3	<0.001 ^a^	40.7 ± 6.4	25.3 ± 6.4	<0.001 ^a^	0.025 ^a^	<0.000 ^a^
			

**Table 2:** Changes in F-wave, M-wave, CSP parameters and Toronto Score between the two timepoints (waning and peak response phase) per study group (ONA and ABO). (a—*t*-test, c—Mann–Whitney).

Parameter	ΔONA Mean ± SD	ΔABO Mean ± SD	*p*-Value
Fmin (ms)	0.6 ± 1.0	1.5 ± 1.2	0.804 ^c^
Fmax (ms)	0.8 ± 2.0	2.3 ± 1.2	0.104 ^c^
Fmean (ms)	2.7 ± 1.3	9.4 ± 0.9	0.227 ^c^
Fchronodisp (ms)	9.3 ± 2.0	5.27 ± 1.5	0.094 ^c^
Fduration (ms)	6.8 ± 1.7	2.5 ± 4.2	0.454 ^c^
Fpersistence (*n*)	1.6 ± 3.1	1.14 ± 1.1	0.578 ^a^
Fampl (µV)	1.1 ± 0.1	1.1 ± 0.1	0.427 ^c^
F/Mampl	6.6 ± 2.7	4.7 ± 1.4	0.903 ^a^
F–Mlat (ms)	0.6 ± 1.1	2.53 ± 1.2	0.603 ^c^
CSPo (ms)	9.8 ± 11.0	1.0 ± 3.1	0.603 ^c^
CSPe (ms)	6.2 ± 8.6	1.4 ± 7.1	0.114 ^c^
CSPd (ms)	4.4 ± 9.4	9.4 ± 6.0	0.200 ^a^
Toronto score	6.3 ± 9.6	2.4 ± 6.3	0.551 ^a^

## Data Availability

The patient data comprising this study’s database is not publicly accessible. This restriction stems from its hosting on the hospital’s private server, which, in accordance with Polish law and GDPR mandates, enforces limitations on external access.

## Abbreviations

The following abbreviations are used in this manuscript:

ABO	abobotulinumtoxinA (Dysport^®^)
APB	abductor pollicis brevis
ACap	anterocaput
BoNT-A	botulinum toxin type A
CD	cervical dystonia
CMAP	compound muscle action potential
CSP	cutaneous silent period
CSPd	cutaneous silent period duration
CSPe	cutaneous silent period end
CSPo	cutaneous silent period onset
EMG	electromyography
GCP	Good Clinical Practice
LCap	laterocaput
NAPs	neurotoxin-associated proteins
ONA	onabotulinumtoxinA (Botox^®^)
PTNK	Polskie Towarzystwo Neurofizjologii Klinicznej [Polish Society of Clinical Neurophysiology]
RCap	retrocaput
ST	sensory threshold
TCap	Torticaput
TWSTRS	Toronto Western Spasmodic Torticollis Rating Scale
